# Linked publications from a single trial: a thread of evidence

**DOI:** 10.1186/1745-6215-15-369

**Published:** 2014-09-23

**Authors:** Douglas G Altman, Curt D Furberg, Jeremy M Grimshaw, Daniel R Shanahan

**Affiliations:** Centre for Statistics in Medicine, University of Oxford, Botnar Research Centre, Windmill Road, Oxford, OX3 7LD UK; Division of Public Health Sciences, Wake Forest University School of Medicine, Medical Center Boulevard, Winston-Salem, NC 27157-1063 USA; Clinical Epidemiology Programme, Ottawa Hospital Research Institute and Department of Medicine, University of Ottawa, 501 Smyth Road, Ottawa, ON K1H 8 L6 Canada; BioMed Central Ltd, 236 Gray’s Inn Road, London, WC1X 8HB UK

## Introduction

*Trials* was launched with the ambition of providing authors with the opportunity to provide all the necessary detail for a true and complete scientific record [1]. It has long pushed for the communication of all outcome measures in health-related randomized controlled trials, as well as varying analyses and interpretations, and in-depth descriptions of what was done and what was learnt. An integral part of this was, of course, the publication of study protocols, which had rarely been possible in paper-based journals [2]. A published protocol establishes precedence, allows more detailed discussion of methodological issues and can be referenced when reporting the main trial results [3].

The increasing publication of study protocols is undoubtedly one of *Trials’* greatest successes [4]. However, despite recent movements towards greater transparency in reporting research, concerns remain regarding the widespread discrepancies between trial publications and what was stated in the original study protocol [5, 6]. Indeed, there is strong evidence that selective outcome reporting, with manipulation of the outcomes and analyses reported, is a common problem in medical research [7]. This serves to highlight the importance of researchers having access to all of the relevant information, to reliably evaluate bias or selective reporting in clinical trials.

However, while *Trials* regularly publishes the results of trials, primary trial reports are still predominantly published elsewhere. Although a results paper may reference a published study protocol, there is nothing to connect that report to subsequent publications; and no link from the protocol itself to the results article. This situation is further complicated by the ever-growing body of literature. A single clinical trial can result in multiple publications: the study protocol and traditional results paper or papers, as well as commentaries, secondary analyses and, eventually, systematic reviews, among others [8].

The advent of trial registration has helped to mitigate, to some extent, the lack of connectivity by providing a unique identifier associated with each trial record. However, while major medical journals typically capture the clinical trial number on or before publication, many do not follow *Trials’* example by publishing it in the abstract, as is recommended by CONSORT for Abstracts [9] and the International Committee of Medical Journal Editors (ICMJE) [10]. The reader thus has to identify related content through a literature search, using titles, authors and the abstract; details that will not necessarily remain consistent, as the articles may be published in different journals, with different authors, over many years.

## Threaded publications

In 1999, Chalmers and Altman envisioned a solution. In their article in *The Lancet*, they wrote: ‘Electronic publication of a protocol could be simply the first element in a sequence of “threaded” electronic publications, which continues with reports of the resulting research (published in sufficient detail to meet some of the criticisms of less detailed reports published in print journals), followed by deposition of the complete data set’ [2]. This was the first description of the threaded publications initiative.

Building on the concept of trial registration, the aim of the threaded publications initiative is to link all publications relating to a single clinical trial centrally, regardless of journal or publisher, using the clinical trial number. This means that no matter which article in the ‘thread’ researchers start from, they will be easily able to identify and gain access to all other publications relating to that clinical trial. In 2011, we began to put this linkage into practice within *Trials* [11]; however, to achieve its fundamental aims, the project must go beyond a single journal or publisher.

It was in pursuit of this ambition that, in partnership with CrossRef, BioMed Central called a cross-publisher meeting to discuss how to build on the well-established digital-object identifier (DOI) and the more recent CrossMark tool, which provides information on the status of the associated article and an additional publication record, to achieve the threaded publications concept [12]. The meeting spurred the formation of a working group to govern the development of the project. Chaired by BioMed Central, the working group includes representatives from CrossRef, *BMJ*, *Canadian Medical Associate Journal*, the Cochrane Collaboration, *eLife*, F1000, the Internal Standard Randomized Controlled Trial Number (ISRCTN) Registry, *The Lancet*, Origin Editorial, the Public Library of Science, Springer, The Wellcome Trust and Wiley, and is overseeing the development of the first phase in the threaded publications concept, Linked Reports of Clinical Trials.

## Linked Reports of Clinical Trials

The Linked Reports of Clinical Trials project will adapt the existing CrossMark standard to capture additional metadata about an article, namely the clinical trial number, the trial registry and the relation to the primary trial report, and associate that information with the article DOI on publication. A query to this CrossRef database will then return all articles related to that clinical trial number. As all the metadata will be open access (CC0 license) [13], with no copyright, it will be possible to access this article ‘thread’ either through the CrossMark interface or independently through an application programming interface (API).

A pilot evaluation of this system is set to begin towards the end of 2014, trialling how to adapt existing article workflows to capture these additional metadata prospectively and in an efficient and sustainable manner. For the initial pilot phase, only those articles for which the clinical trial number is currently required for publication by the publishers involved will be included; this includes study protocols [14] and updates [15], statistical analysis plans [16], primary results papers [17] and secondary analyses [18]. Following successful implementation on this subset, the scope will be expanded to capture the clinical trial number for additional article types related to clinical trials, such as case reports of adverse events, commentaries and editorials.

## Conclusion

The medical literature is vast and it is impossible to keep up with the deluge of new research articles. With ongoing concerns regarding manipulation of the outcomes and analyses reported in medical research, it is increasingly important that researchers are able to easily identify and access all publications relating to a specific clinical trial, in order to obtain the complete picture and to evaluate bias or selective reporting reliably. Recent developments and innovations within the threaded publications initiative and the Linked Reports of Clinical Trials project demonstrate progress towards the ideal of making all trial information readily available.

## Abbreviations

CONSORT: Consolidated Standards of Reporting Trials
DOI: Digital-object identifier
ICMJE: International Committee of Medical Journal Editors
ISRCTN: Internal Standard Randomized Controlled Trial Number
API: Application programming interface.
